# The presence of anti-Tat antibodies in HIV-infected individuals is associated with containment of CD4^+^ T-cell decay and viral load, and with delay of disease progression: results of a 3-year cohort study

**DOI:** 10.1186/1742-4690-11-49

**Published:** 2014-06-24

**Authors:** Stefania Bellino, Antonella Tripiciano, Orietta Picconi, Vittorio Francavilla, Olimpia Longo, Cecilia Sgadari, Giovanni Paniccia, Angela Arancio, Gioacchino Angarano, Nicoletta Ladisa, Adriano Lazzarin, Giuseppe Tambussi, Silvia Nozza, Carlo Torti, Emanuele Focà, Guido Palamara, Alessandra Latini, Laura Sighinolfi, Francesco Mazzotta, Massimo Di Pietro, Giovanni Di Perri, Stefano Bonora, Vito S Mercurio, Cristina Mussini, Andrea Gori, Massimo Galli, Paolo Monini, Aurelio Cafaro, Fabrizio Ensoli, Barbara Ensoli

**Affiliations:** 1 National AIDS Center, Istituto Superiore di Sanità, Rome, Italy; 2 Pathology and Microbiology, San Gallicano Institute, Istituti Fisioterapici Ospitalieri, Rome, Italy; 3 Division of Infectious Diseases, University of Bari, Policlinic Hospital, Bari, Italy; 4 Division of Infectious Diseases, S. Raffaele Hospital, Milan, Italy; 5 Division of Tropical and Infectious Diseases, Spedali Civili, Brescia, Italy; 6 Department of Infectious Dermatology, San Gallicano Hospital, Rome, Italy; 7 Unit of Infectious Diseases, University Hospital of Ferrara, Ferrara, Italy; 8 Unit of Infectious Diseases, S.M. Annunziata Hospital, Florence, Italy; 9 Amedeo di Savoia Hospital, Turin, Italy; 10 Department of Infectious Diseases, S. Maria Goretti Hospital, Latina, Italy; 11 Division of Infectious Diseases, University Policlinic of Modena, Modena, Italy; 12 Division of Infectious Diseases, San Gerardo Hospital, University of Milan Bicocca, Monza, Italy; 13 Institute of Tropical and Infectious Diseases, L. Sacco Hospital, University of Milan, Milan, Italy

**Keywords:** HIV progression, Tat, Antibodies, CD4^+^ T cells, Viral load

## Abstract

**Background:**

Tat is a key HIV-1 virulence factor, which plays pivotal roles in virus gene expression, replication, transmission and disease progression. After release, extracellular Tat accumulates in tissues and exerts effects on both the virus and the immune system, promoting immune activation and virus spreading while disabling the host immune defense. In particular, Tat binds Env spikes on virus particles forming a virus entry complex, which favors infection of dendritic cells and efficient transmission to T cells via RGD-binding integrins. Tat also shields the CCR5-binding sites of Env rendering ineffective virus neutralization by anti-Env antibodies (Abs). This is reversed by the anti-Tat Abs present in natural infection or induced by vaccination.

**Findings:**

Here we present the results of a cohort study, showing that the presence of anti-Tat Abs in asymptomatic and treatment-naïve HIV-infected subjects is associated with containment of CD4^+^ T-cell loss and viral load and with a delay of disease progression. In fact, no subjects with high anti-Tat Ab titers initiated antiretroviral therapy during the three years of follow-up. In contrast, no significant effects were seen for anti-Env and anti-Gag Abs. The increase of anti-Env Ab titers was associated with a reduced risk of starting therapy only in the presence of anti-Tat Abs, suggesting an effect of combined anti-Tat and anti-Env Abs on the Tat/Env virus entry complex and on virus neutralization.

**Conclusions:**

Anti-Tat immunity may help delay HIV disease progression, thus, targeting Tat may offer a novel therapeutic intervention to postpone antiretroviral treatment or to increase its efficacy.

## Findings

The HIV-1 Tat protein plays essential roles in the virus life cycle and in pathogenesis [1-9], representing a key HIV virulence factor. Tat is produced very early upon infection [1-5] and is released extracellularly [1,4,5]. By binding to heparan sulfate proteoglycans with its basic region, extracellular Tat accumulates in tissues [4] where it exerts effects on both the virus and the immune system [1-11], making it an optimal target for an immune intervention based on antibody (Ab) responses [12-14]. In particular, extracellular Tat activates virus and cellular gene expression and replication, increasing virus transmission to neighbor cells [1,6-9,15,16]. Further, extracellular Tat binds Env spikes forming a virus entry complex that favors infection of dendritic cells (DC) and efficient transmission to T cells, key target cells in primary infection that will later constitute the virus reservoir [16]. This occurs by redirecting virus entry from the canonical receptors to RGD-binding integrins that Tat uses as receptors to enter DC and other cells of the reticular-endothelial cell system [16]. Of note, by binding the Env CCR5 co-receptor binding sites, Tat shields Env from anti-HIV Abs, thus inhibiting virus neutralization by HIV sera, which, however, can be restored and further increased by anti-Tat Abs either present in natural infection or induced by vaccination [16]. Notably, in natural HIV infection_,_ anti-Tat Abs are produced by only a small fraction of individuals [17,18], while, in contrast, high Ab titers are produced against all other viral products [19]. The reason for such a limited anti-Tat Ab response is unclear. However, Tat has potent immunoregulatory functions [10,11] and its capacity to target, enter and induce DC maturation toward a prevalent Th1 response [10,11] may have implications for the setting of the anti-HIV-1 immune response and in AIDS pathogenesis. In fact, when present, anti-Tat Abs correlate with the asymptomatic state and lower disease progression [20-24]. In particular, a higher prevalence of anti-Tat Abs has been shown in asymptomatic and in non-progressors HIV-1-infected individuals as compared to patients in advanced disease or to fast progressors [20-24]. A cross-sectional and longitudinal study, on 252 HIV-1 seroconverters, with a median follow-up time of 7.2 years, indicated that the presence of anti-Tat Abs is predictive of a slower progression to AIDS or immunodeficiency [21]. Progression was faster in persistently anti-Tat Ab-negative than in transiently anti-Tat Ab-positive subjects, whereas no progression was observed in individuals persistently anti-Tat Ab-positive [21]. On the other hand, Tat vaccination in monkeys can prevent or control infection with pathogenic SHIV [25], and this correlates with Tat-specific Abs [12,26]. Thus, anti-Tat Abs may represent a predictive biomarker of a slower progression to AIDS.

The effects of anti-Tat Abs on the immunological, virological and clinical outcome of HIV-infected subjects were assessed in a prospective observational study (ISS OBS T-003, ClinicalTrials.gov NCT01029548) conducted in asymptomatic drug-naïve HIV-infected adult volunteers enrolled in eight clinical centers in Italy. The study was approved by the local Ethics Committees and all patients signed the informed consent prior to enrollment. The study population consisted of 61 individuals, with CD4^+^ T-cell counts ≥400/μl and levels of plasma viremia ≤100,000 copies/ml, enrolled between 2008 and 2011, and followed for 42 months with visits every 3 months. The median follow-up was 24 months. The characteristics at baseline of the study participants are shown in Table 1.

**Table 1:** Characteristics at baseline of the study participants

	**All subjects**	**Anti-Tat Ab-positive**^**a**^	**Anti-Tat Ab-negative**
	**(*n*** **= 61)**	**(*n*** **= 20)**	**(*n*** **= 41)**
Age (years)	38 (32–42)	38 (32–43)	38 (32–41)
Male (%)	90.2	95.0	87.8
Female (%)	9.8	5.0	12.2
CD4^+^ (cells/μl)	544 (463–678)	546 (500–702)	541 (454–640)
CD4^+^ (%)	29.0 (25.0-34.0)	29.0 (25.0-33.0)	28.5 (25.5-34.5)
Viral load (log_10_ copies/ml)	4.2 (3.7-4.5)	4.2 (3.7-4.6)	4.2 (3.9-4.4)
Years from diagnosis of HIV^b^	1.3 (0.9-3.2)	1.0 (0.7-4.0)	1.6 (1.0-2.7)
HAART initiation since HIV^+^ (years)^c^	3.6 (2.9-5.0)	3.6 (3.2-5.4)	3.5 (2.6-5.0)
HAART initiation since study entry (months)^c^	22 (14–19)	30 (28–31)	17 (13–22)

Data are expressed as median with interquartile range (IQR).

^a^Eight individuals became anti-Tat Ab-positive during follow-up; ^b^Based on 52 individuals.

^c^Based on data from 13 individuals, 4 anti-Tat Ab-positive with low and transient anti-Tat Abs (3 IgM and 1 IgG) and 9 anti-Tat Ab-negative.

Determination of anti-Tat Abs and plasma viral load were performed by a centralized laboratory [13,14]. Anti-Tat Abs as well as anti-Env and anti-Gag IgG were assessed by ELISA as previously described [19,21], using 100 ng of Tat or gp120 or p55 Gag/well, respectively. Ab titers equal or higher than 25 for IgM and IgA, or 100 for IgG were considered positive.

HIV-1 viral load was determined with the COBAS AmpliPrep/COBAS TaqMan HIV-1 Test, version 2.0. CD4^+^ T-cell counts were performed at each clinical site according to standard national laboratory measurements. Statistical analyses were carried out at two-sided with a 0.05 significance level, using SAS^®^ software, version 9.2.

Of the 61 subjects enrolled in the study, 20 (32.8%) were anti-Tat Ab-positive. Eleven of these had median titers of 600 for IgG, 25 for IgM and 100 for IgA, which persisted for the entire study (Table 2). These individuals were termed “high” anti-Tat Ab-positive subjects. The other 9 patients had anti-Tat Ab titers of 100 for IgG or 25 for IgM, and no IgA (Table 2); 4 of these subjects had persistent Abs, whereas 5 had transient Abs (alternately negative and positive). These were defined as “low” anti-Tat Ab-positive subjects. Twelve subjects were anti-Tat Ab-positive at study entry, while 8 individuals developed Abs during follow-up (between months 3 and 21), 2 patients with high and 6 with low anti-Tat Abs, respectively. The median anti-Env and anti-Gag IgG titers were higher in high anti-Tat Ab-positive subjects as compared to individuals with low or no Abs (Table 2). The baseline demographic and clinical characteristics were comparable between subjects with or without anti-Tat Abs (Table 1).

**Table 2:** **Anti-Tat, anti-Env and anti-Gag antibody responses**.

	**High anti-Tat Ab titers**	**Low anti-Tat Ab titers**	**No anti-Tat Ab**
	**(*n*** **= 11)**	**(*n*** **= 9)**	**(*n*** **= 41)**
**Anti-Tat Ab-positive (n, %)**			
IgM and IgG positive	3 (27%)	0 (0%)	0 (0%)
IgG and IgA positive	1 (9%)	0 (0%)	0 (0%)
IgM positive	0 (0%)	6 (67%)	0 (0%)
IgG positive	6 (55%)	3 (33%)	0 (0%)
IgA positive	1 (9%)	0 (0%)	0 (0%)
**Anti-Env Ab-positive (n, %)**	11 (100%)	9 (100%)	41 (100%)
**Anti-Gag Ab-positive (n, %)**	11 (100%)	9 (100%)	41 (100%)
**Median titers (range)**			
IgM anti-Tat	25 (25–25)	25 (25–25)	<25
IgG anti-Tat	600 (200–12800)	100 (100–100)	<100
IgA anti-Tat	100 (25–200)	<25	<25
IgG anti-Env	12,800 (800–51,200)	3,200 (400–12,800)	6,400 (800–38,400)
IgG anti-Gag	102,400 (4,000-2,457,600)	12,800 (1,600-819,200)	19,200 (200–3,276,800)

**Figure 1:**
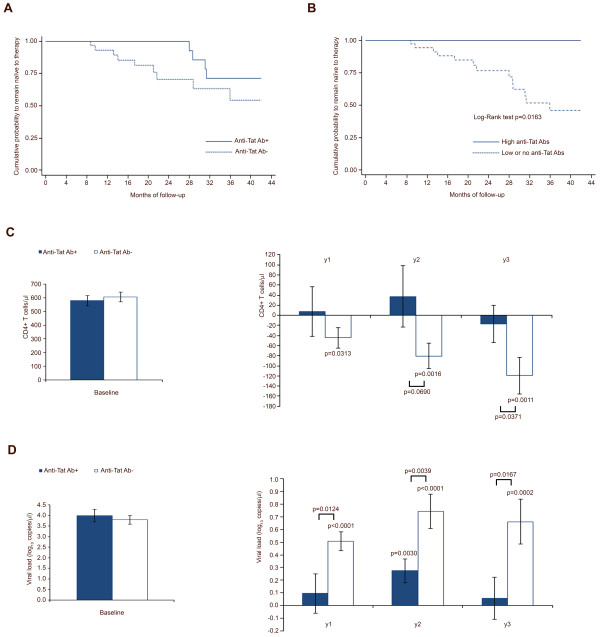
**Kaplan-Meier curves, CD4**^**+ **^**T-cell number and viral load stratified by anti-Tat Abs. (A)** Cumulative probability to remain naïve to therapy according to the presence (n = 20) or absence (n = 41) of anti-Tat Abs, and **(B)** for subjects with high titers of anti-Tat Abs (n = 11) versus subjects with low/no anti-Tat Abs (n = 50). **(C)** Baseline values and changes from baseline values of CD4^+^ T-cell counts and **(D)** viral load levels at years 1, 2 and 3, according to the presence or absence of anti-Tat Abs, respectively, in subjects naïve to therapy (anti-Tat Ab-positive n = 16 year 1, n = 10 year 2, n = 10 year 3; anti-Tat Ab-negative n = 32 year 1, n = 17 year 2, n = 9 year 3). A longitudinal analysis for data arising from repeated measures, adjusted for baseline values, was applied, using the generalized estimating equations method, where the measurements were assumed to be multivariate normal. Data are presented as mean values with standard error.

Of the 61 subjects, 13 started the highly active antiretroviral therapy (HAART) during follow-up, 1 for concomitant diseases, 1 for high viral load, 4 for low CD4^+^ T-cell counts and 7 for both low CD4^+^ T cells and high viral load. The median values of CD4^+^ T cells and viral load at HAART initiation were 364 cells/μl and 136,000 copies/ml, respectively. Of these patients, 9 were anti-Tat Ab-negative at study entry and remained negative during follow-up, while the other 4 patients had low and transient anti-Tat Abs (3 IgM and 1 IgG) since month 15 or 18 of follow-up, respectively. Conversely, none of the 11 subjects with high anti-Tat Abs started HAART. The cumulative probability to remain naive to therapy was higher in the anti-Tat Ab-positive than in the anti-Tat Ab-negative subjects (Figure 1A), and this difference was statistically significant when subjects with high anti-Tat Ab titers were compared with individuals with no or low anti-Tat Ab titers (Figure 1B). In particular, high anti-Tat Ab-positive subjects showed no progression for the entire follow-up (42 months), whereas the median time to HAART was 30 months and 17 months for patients with low or no anti-Tat Abs, respectively.

The risk of starting therapy was 84% lower in anti-Tat Ab-positive subjects as compared to anti-Tat Ab-negative individuals. No effects were observed for anti-Gag IgG titers, whereas 81% lower risk was detected with the increase of anti-Env IgG titers, but only in the presence of anti-Tat Abs, and the effect was lost in anti-Tat Ab-negative subjects (Table 3).

**Table 3:** Risk of starting antiretroviral therapy

**Parameters**	**Hazard ratio**	**95% confidence limits**	**P-value**
*All subjects (n = 52)*			
Anti-Tat Ab + vs Anti-Tat Ab-	0.16	0.03 – 0.84	0.0305
IgG anti-Env (log_10_ titers)	0.19	0.05 – 0.73	0.0148
IgG anti-Gag (log_10_ titers)	0.78	0.41 – 1.48	0.4573
Years from diagnosis of HIV	1.27	0.91 – 1.76	0.1631
CD4^+^ T cells/μl at baseline	1.00	0.99 – 1.00	0.3309
Viral load (log_10_ copies/ml) at baseline	2.53	0.75 – 8.52	0.1344
*Anti-Tat Ab-negative subjects (n = 35)*			
IgG anti-Env (log_10_ titers)	0.41	0.06 – 2.67	0.3545
IgG anti-Gag (log_10_ titers)	1.57	0.27 – 9.04	0.6101
Years from diagnosis of HIV	1.00	0.46 – 2.20	0.9935
CD4^+^ T cells/μl at baseline	0.99	0.98 – 1.01	0.3445
Viral load (log_10_ copies/ml) at baseline	24.20	0.22 – 2671.23	0.1843

A Cox proportional hazards model with time-dependent repeated measurements was used to estimate the effects of the presence of anti-Tat Abs, or of anti-Env or anti-Gag IgG titers on the risk of starting HAART, after adjusting for years from HIV diagnosis, CD4^+^ T cells and viral load at baseline. Anti-Env and anti-Gag Abs were assessed at baseline and at 6, 18 and 36 months.

A longitudinal analysis of the yearly changes of CD4^+^ T-cell counts and viral load was then performed for the 48 subjects who remained naive to therapy during the study. The results showed a significant containment of CD4^+^ T-cell loss and of plasma viral load increases in anti-Tat Ab-positive subjects during the three years of follow-up, as compared to anti-Tat Ab-negative subjects (Figure 1C-D).

**Figure 2:**
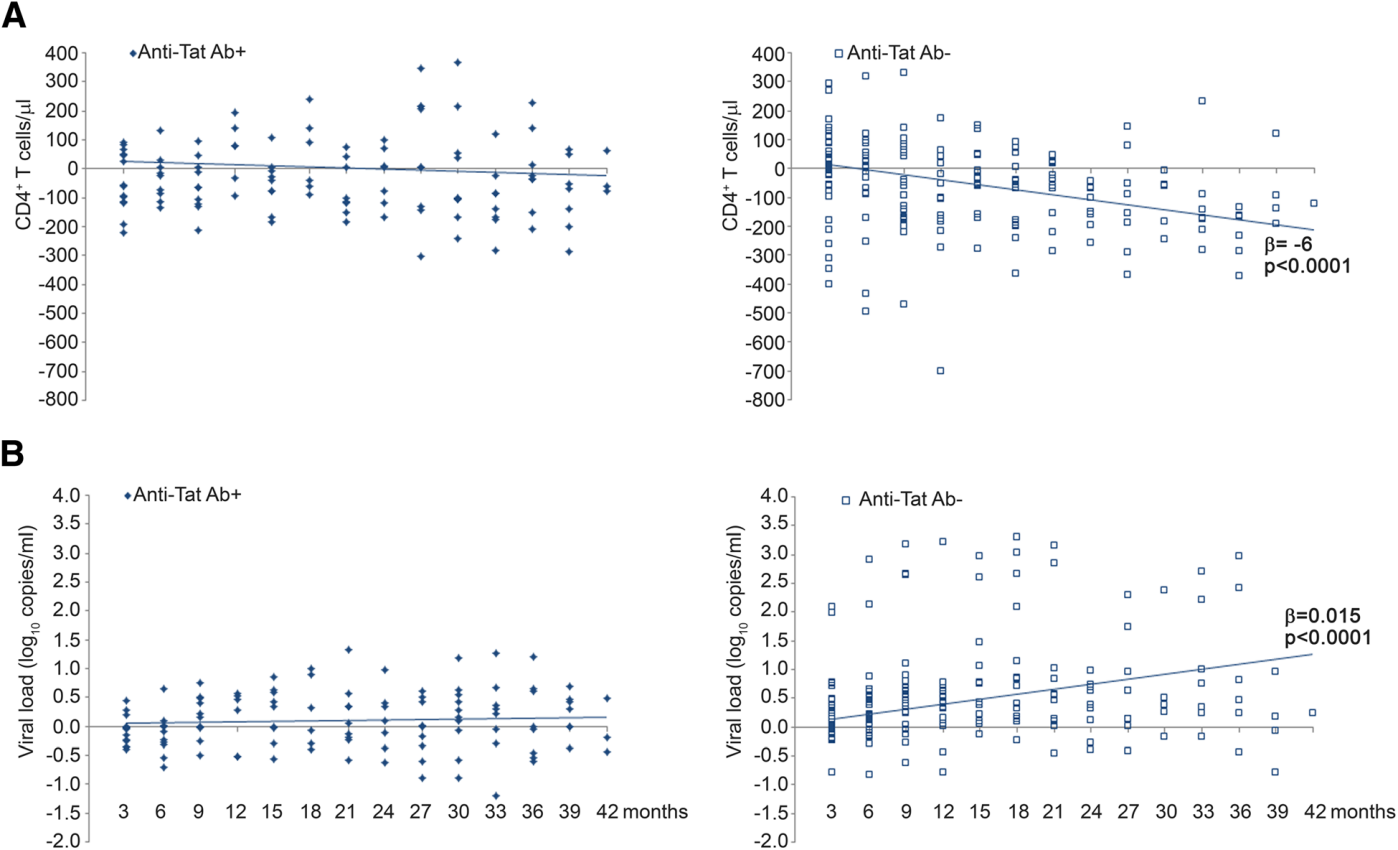
**Changes over time of CD4**^**+ **^**T-cell number and viral load by anti-Tat Abs in individuals naïve to therapy. (A)** CD4^+^ T-cell counts and **(B)** viral load in subjects who remained naive to therapy were analyzed over time, according to the presence or absence of anti-Tat Abs, by applying a random-effect regression model. The decrease from baseline of CD4^+^ T cells/μl was -1.1 (95% CI -3.7; 1.5) per month in the anti-Tat Ab-positive subjects and -5.9 (95% CI -8.7; -3.1, p < 0.0001) per month in the anti-Tat Ab-negative individuals, respectively. The difference between the coefficients of regression was statistically significant (p = 0.0060). Similarly, the increase of viral load was 0.003 log_10_ copies/ml (95% CI -0.004; 0.010) per month in anti-Tat Ab-positive patients and 0.015 (95% CI 0.009; 0.022, p < 0.0001) per month in anti-Tat Ab-negative subjects, respectively. The difference between the slopes was statistically significant (p = 0.0105). All longitudinal samples from 48 individuals were included in the analysis.

CD4^+^ T-cell counts and viral load were also analyzed over time by applying a random coefficient model, showing for anti-Tat Ab-negative individuals a decrease of 6 CD4^+^ T-cells/μl and an increase of 3.6% of viral load per month, respectively, while no significant changes were detected in anti-Tat Ab-positive subjects (Figure 2A-B). When anti-Env and anti-Gag Abs were included in the analysis, the protective role of the presence of the anti-Tat Abs remained, while no significant effects were observed for anti-Env or anti-Gag Abs (Table 4).

**Table 4:** **Changes from baseline of CD4**^**+ **^**T cells and viral load by a longitudinal analysis.**

**Parameters**	**Estimate**	**95% confidence limits**	**P-value**
**CD4**^**+ **^**T cells/μl**			
Change per month in Anti-Tat Ab+	-1.6	-6.2; 3.0	0.4768
Change per month Anti-Tat Ab–	-8.9	-13.8; -3.9	0.0011
IgG anti-Env (log_10_ titers) anti-Tat Ab+	133.4	-130.0; 396.8	0.3070
IgG anti-Env (log_10_ titers) anti-Tat Ab–	150.0	-40.7; 340.6	0.1178
IgG anti-Gag (log_10_ titers) anti-Tat Ab+	-15.6	-141.6; 110.5	0.8014
IgG anti-Gag (log_10_ titers) anti-Tat Ab–	-56.0	-177.8; 65.9	0.3533
**Viral load (log**_**10 **_**copies/μl)**			
Change per month in Anti-Tat Ab+	0.005	-0.007; 0.018	0.3898
Change per month Anti-Tat Ab–	0.011	0.001; 0.021	0.0401
IgG anti-Env (log_10_ titers) anti-Tat Ab+	0.26	-0.64; 1.16	0.5560
IgG anti-Env (log_10_ titers) anti-Tat Ab–	0.08	-0.67; 0.83	0.8249
IgG anti-Gag (log_10_ titers) anti-Tat Ab+	-0.19	-0.57; 0.18	0.3006
IgG anti-Gag (log_10_ titers) anti-Tat Ab–	-0.15	-0.55; 0.26	0.4632

Multivariate analysis for repeated measures of longitudinal samples from 41 individuals (random-effect regression model). Anti-Env and anti-Gag Abs were assessed at baseline and at 6, 18 and 36 months.

The results of the present study indicate a significant association between the presence of anti-Tat Abs and a slower disease progression. The increase of anti-Env IgG titers was associated with a lower risk of starting HAART only in the presence of anti-Tat Abs, suggesting that anti-Tat and anti-Env Ab combined have increased HIV neutralizing effects by blocking the Tat/Env complex formation and virus entry, as shown earlier both in vitro and in vivo [16,26]. Thus both anti-Tat and anti-Env Abs appear to be required to efficiently counteract HIV disease progression. In contrast, no significant effects of anti-Env or anti-Gag Ab titers were observed on CD4^+^ T-cell counts and viral load in patients naive to therapy with or without anti-Tat Abs. Overall, anti-Tat Ab-positive patients showed a remarkable preservation of CD4^+^ T cells and containment of viral load for the entire follow-up (3 years), and no individuals with high levels of anti-Tat Abs initiated HAART during follow-up.

Based on this notion, phase I-II trials of therapeutic immunization with the Tat protein have been conducted in HIV-infected asymptomatic or HAART-treated patients in Italy (ISS T-001, ClinicalTrials.gov NCT00505401 and ISS T-002 ClinicalTrials.gov NCT00751595, respectively) [13,14] and a phase II trial is ongoing in South Africa in patients under HAART (ISS T-003, ClinicalTrials.gov NCT01513135). The results from these studies indicate that the Tat vaccine is safe and immunogenic, since it induced high titers of anti-Tat Abs with a remarkable persistence over time and with characteristics similar to those of the subjects with high anti-Tat Abs described here. More importantly, Tat vaccination in HAART-treated subjects increased CD4^+^ T-cell number, restored functional CD4^+^ and CD8^+^ subsets (i.e. central memory T cells), increased B and NK cell numbers, and progressively reduced HIV proviral DNA [14], *Ensoli F. et al., manuscript submitted*]. These data suggest that the induction of an anti-Tat humoral immune response may effectively help delay, and possibly counteract HIV disease progression and antiretroviral treatment initiation.

### Ethical approved

The study was approved by the following local Ethics Committees: Policlinic of Bari, Bari, Italy; Fondazione S. Raffaele, Milan, Italy; Spedali Civili, Brescia, Italy; Istituti Fiosterapici Ospitalieri San Gallicano, Rome, Italy; Arcispedale S. Anna, Ferrara, Italy; S.M. Annunziata Hospital, Florence, Italy; Amedeo di Savoia Hospital, Turin, Italy; S. Maria Goretti Hospital, Latina, Italy; Policlinic of Modena, Modena, Italy; Azienda Ospedaliera San Gerardo, Monza, Italy; L. Sacco Hospital, Milan, Italy.

## Competing interests

The authors declare no competing financial interests.

## Authors’ contributions

SB and AT equally contributed to the manuscript preparation and to the interpretation of the data (in particular, SB performed statistical analyses and AT supervised the laboratory work). OP contributed to the manuscript preparation and supervised the clinical study data management. VF, GP and AA contributed to the study with the laboratory testing. OL and CS supervised the clinical study management and contributed to manuscript preparation. GA, NL, AL, GT, SN, CT, EF, GP, AL, LS, FM, MDP, GDP, SB, VSM, CM, AG and MG conducted the study at the clinical sites. PM contributed to the manuscript critical review. AC and FE contributed to the manuscript preparation, interpretation of the data and supervised the experimental work. BE conceived and designed the study, supervised the experimental work, data analysis and interpretation, and manuscript preparation. All authors read and approved the final manuscript.
